# The complete chloroplast genome of *Viola mandshurica* (Violaceae) and its phylogenetic analysis

**DOI:** 10.1080/23802359.2026.2699499

**Published:** 2026-07-08

**Authors:** Feng Ni, Lina Pan, Jingjing Xia, Dujuan Zhan

**Affiliations:** a Modern Industry College of Traditional Chinese Medicine and Health, Lishui University, Lishui, China; b Huayong Engineering Design Group Co., Ltd, Ningbo, China; c Ningbo Hangwan Real Estate Co., Ltd, Ningbo, China; d College of Agriculture and Biotechnology, Lishui University, Lishui, China

**Keywords:** Organellar DNA, genomic resource, phylogenetic relationship, Illumina sequencing

## Abstract

*Viola mandshurica* is a perennial herb of Violaceae. In this study, we reported and characterized the complete chloroplast genome of *V. mandshurica* based on Illumina paired-end sequencing data. The complete chloroplast genome was 156,476 bp in length, with an overall GC content of 36.30%. A total of 112 unique genes were annotated, comprising 78 protein-coding genes, 30 tRNA genes, and four rRNA genes. Phylogenetic analysis showed that *V. mandshurica* was placed within the genus *Viola*. This chloroplast genome provides valuable genomic information for further studies on species identification, phylogeny, and plastome evolution in Violaceae.

## Introduction

*Viola mandshurica* W. Becker 1917 is a perennial herbaceous species of Violaceae, native to the Russian Far East, northern and eastern China, and Japan, where it mainly occurs in temperate regions. The genus *Viola* is one of the largest genera in Violaceae, comprising approximately 664 accepted species and showing substantial morphological diversity and complex infrageneric relationships (Marcussen et al. 2022). Because many *Viola* species are morphologically similar and taxonomically difficult to distinguish, additional genomic resources are useful for species identification and for improving phylogenetic resolution within the genus.

Chloroplast genomes have been widely used in plant systematics, species identification, and evolutionary studies because of their conserved genome structure, mostly uniparental inheritance, and informative sequence variation. Several *Viola* plastomes have been reported, and these data have helped clarify relationships among closely related species (Cheon et al. 2019; Cao et al. 2022; Moon and Kim 2023). However, the complete chloroplast genome of *V. mandshurica* has not been sufficiently characterized, and its placement based on plastome-scale data remains poorly documented.

In this study, we sequenced, assembled, and annotated the complete chloroplast genome of *V. mandshurica*. We characterized its genome structure, gene content, and splicing genes, and reconstructed its phylogenetic relationship with related *Viola* species using complete chloroplast genome sequences. This study provides a new plastome resource for *V. mandshurica* and contributes to future studies of species identification, comparative plastomics, and phylogenetic relationships in *Viola* and Violaceae.

## Materials and methods

**Figure 1.**
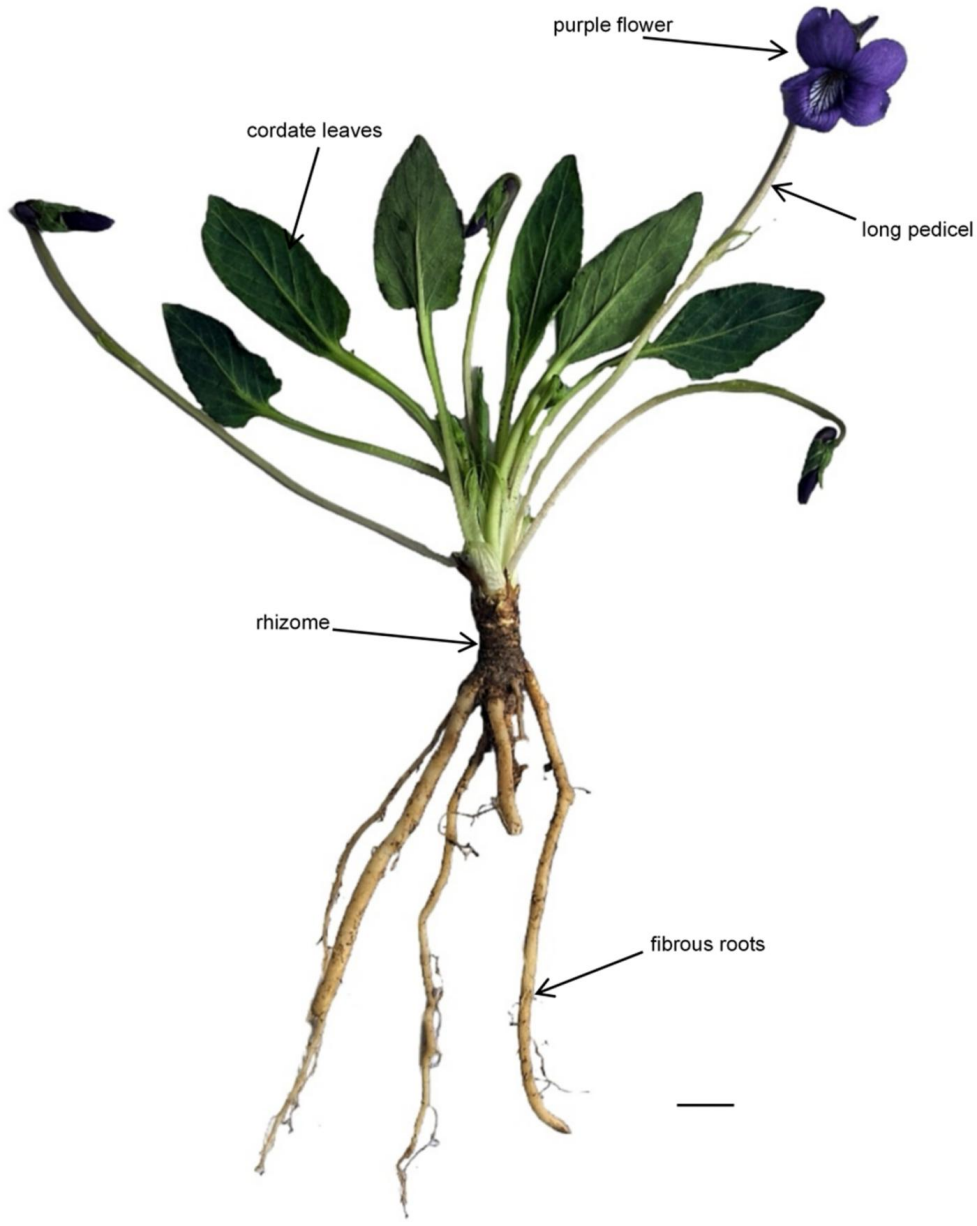
Morphological features of *Viola mandshurica*. Whole plant photographed immediately after collection and prior to specimen pressing. Labels indicate selected diagnostic characters, including purple flower, long pedicel, cordate leaves, rhizome, and fibrous roots. Photo credit: Feng Ni. Scale bar = 1 cm.

Fresh leaves of *V. mandshurica* were collected from Mount Tai, Tai’an, Shandong Province, China (36°16′00″N, 117°06′00″E). The specimen was identified by Feng Ni based on morphological characteristics. A voucher specimen was formally deposited in the Herbarium of Lishui University, Lishui University, Lishui, Zhejiang, China, under the provisional institutional herbarium acronym LISHUI and accession number LISHUI-2026-03-015 (contact person: Dujuan Zhan, zhandujuan@lsu.edu.cn; Figure 1).

Total genomic DNA was isolated from leaf tissue with the Rapid Plant Genomic DNA Isolation Kit (Sangon Biotech, Shanghai, China). Paired-end sequencing was then performed on the Illumina HiSeq 2500 platform, generating 19,557,228 raw reads (9,778,614 paired-end read pairs) of 150 bp. After quality filtering, 18,837,392 clean reads were retained. The chloroplast genome was de novo assembled using GetOrganelle v1.8.0.1 with the embryophyte plastome seed database (embplant_pt, version 0.0.1), 15 extension rounds, and SPAdes k-mer sizes of 21, 45, 65, 85, and 105 (Jin et al. 2020). The assembly was circularized automatically by GetOrganelle. Clean reads were mapped back to the assembled plastome, and mapping statistics and per-site sequencing depth were calculated using SAMtools v1.16.1 (Li et al. 2009). A total of 2,320,381 primary reads mapped to the plastome, providing 100% genome coverage with a mean sequencing depth of 2205.45× and a depth range of 490–3412×; the coverage distribution was plotted in R with the ggplot2 package (Wickham 2016; Figure S1). Genome annotation was initially conducted with GeSeq (Tillich et al. 2017) and CPGAVAS2 (Shi et al. 2019). The two annotation outputs were compared and manually checked. Discrepancies were resolved by comparison with homologous chloroplast genes from closely related *Viola* plastomes and by checking gene boundaries, reading frames, and intron/exon structures. For protein-coding genes, start and stop codons were manually inspected. CPGView (Liu et al. 2023) was used to visualize the plastome map and inspect the structures of cis- and trans-spliced genes.

To determine the phylogenetic position of *V. mandshurica* within *Viola*, 24 representative complete chloroplast genome sequences of *Viola* species were selected from GenBank, with priority given to published complete plastomes and species closely related to *V. mandshurica*. The sampling was intended to clarify the phylogenetic placement of *V. mandshurica* rather than to include every available *Viola* chloroplast genome. *Melicytus ramiflorus* (MW238813; Violaceae) was selected as the outgroup because it belongs to Violaceae and provides an appropriate close outgroup for rooting the *Viola* phylogeny. The complete chloroplast genome sequences were aligned using MAFFT v7 with the –auto option (Katoh and Standley 2013). Ambiguously aligned regions were removed using trimAl v1.5 (Capella-Gutiérrez et al. 2009) with the automated1 option. The final trimmed alignment was 153,805 bp in length and contained 12,007 variable sites and 3,046 parsimony-informative sites. Maximum-likelihood phylogenetic analysis was performed using IQ-TREE 2 (Minh et al. 2020). The best-fit nucleotide substitution model was selected by ModelFinder (Kalyaanamoorthy et al. 2017) according to the Bayesian information criterion (BIC), and TVM+F + R3 was selected as the optimal model. Branch support was assessed with 1000 ultrafast bootstrap replicates. The phylogenetic tree was visualized using the ggtree package (Xu et al. 2022) in R. An additional ML tree was reconstructed from a concatenated supermatrix of 68 shared chloroplast protein-coding genes to assess the robustness of the phylogenetic placement (Figure S2).

## Results

**Figure 2.**
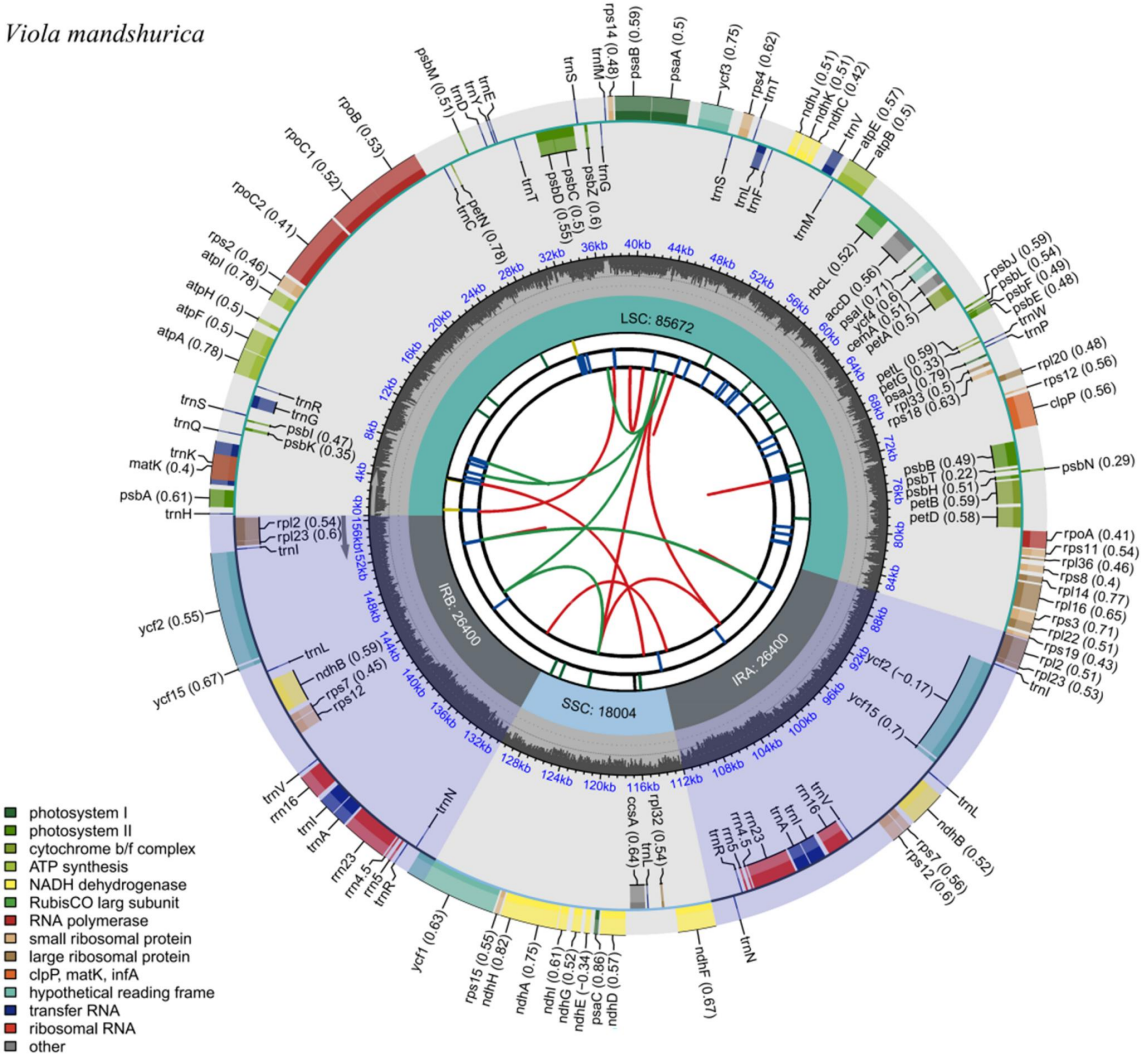
Chloroplast genome map of *Viola mandshurica*. The Central ring shows the relative proportions of GC and AT content, indicated in dark and light grey, respectively. Genes displayed around the outer circle are distinguished by color according to their functional groups.

The complete chloroplast genome of *V. mandshurica* is 156,476 bp in length, with an overall GC content of 36.30%. It exhibits the typical quadripartite plastome structure, comprising an 85,672 bp large single-copy (LSC) region, a pair of 26,400 bp inverted repeat regions (IRa and IRb), and an 18,004 bp small single-copy (SSC) region (Figure 2). Genome annotation revealed 112 unique genes, including 78 protein-coding genes (PCGs), 30 transfer RNA genes, and 4 ribosomal RNA genes. A comparison with nine representative *Viola* plastomes showed that *V. mandshurica* has a genome size, GC content, LSC/SSC/IR lengths, quadripartite structure, and gene content comparable to those of closely related *Viola* species, particularly *V. seoulensis*, *V. prionantha*, and *V. patrinii* (Table S1). Cis-splicing analysis identified ten unique cis-spliced genes, namely *atpF*, *rpoC1*, *ycf3*, *clpP*, *petB*, *petD*, *rpl16*, *rpl2*, *ndhB*, and *ndhA*; among these, *rpl2* and *ndhB* are duplicated in the IR regions (Figure S3). In addition, *rps12* showed a typical trans-spliced structure, with exon 1 located in the LSC region and duplicated exon 2/exon 3 copies positioned in the IR regions (Figures 2 and S4).

**Figure 3.**
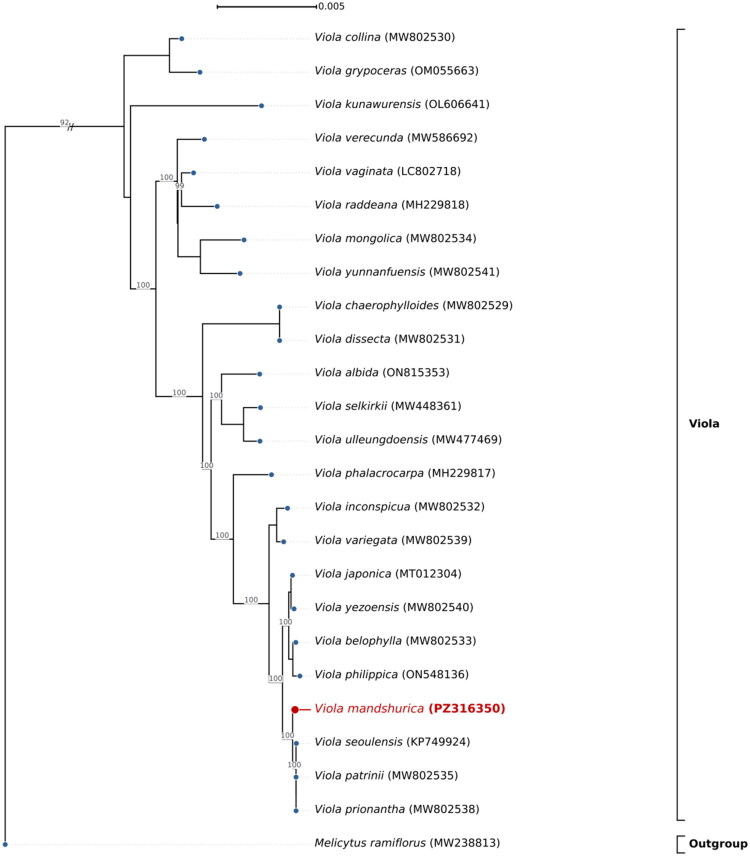
Maximum-likelihood phylogenetic tree of *Viola mandshurica* and related species based on chloroplast genome sequences. *Melicytus ramiflorus* (MW238813) was used as the outgroup. Numbers at nodes represent ultrafast bootstrap support values based on 1000 replicates, and only values ≥70% are shown. The newly sequenced *V. mandshurica* (PZ316350) is highlighted in red. The double slash indicates that the branch leading to the outgroup was shortened for visualization. Scale bar represents substitutions per site. The following sequences were used: *V. kunawurensis* OL606641 (Zhou et al. 2022), *V. collina* MW802530 (Cao et al. 2022), *V. grypoceras* OM055663 (Park et al. 2023), *V. yunnanfuensis* MW802541 (Cao et al. 2022), *V. mongolica* MW802534 (Cao et al. 2022), *V. verecunda* MW586692 (Kwak 2021), *V. raddeana* MH229818 (Cheon et al. 2019), *V. vaginata* LC802718 (Takahashi et al. 2025), *V. chaerophylloides* MW802529 (Cao et al. 2022), *V. dissecta* MW802531 (Cao et al. 2022), *V. albida* ON815353 (Moon and Kim 2023), *V. selkirkii* MW448361 (Go et al. 2022), *V. ulleungdoensis* MW477469 (Go and Yoo 2023), *V. phalacrocarpa* MH229817 (Cheon et al. 2019), *V. inconspicua* MW802532 (Cao et al. 2022), *V. variegata* MW802539 (Cao et al. 2022), *V. mandshurica* PZ316350 (this study), *V. seoulensis* KP749924 (Cheon et al. 2017), *V. prionantha* MW802538 (Cao et al. 2022), *V. patrinii* MW802535 (Cao et al. 2022), *V. philippica* ON548136 (Cao et al. 2022), *V. belophylla* MW802533 (Cao et al. 2022), *V. yezoensis* MW802540 (Cao et al. 2022), *V. japonica* MT012304 (Cheon et al. 2020), and *Melicytus ramiflorus* MW238813 (Maurin et al. 2022).

The maximum-likelihood phylogenetic tree showed that all sampled *Viola* species formed a well-supported clade distinct from the outgroup *M. ramiflorus*. The newly sequenced *V. mandshurica* was firmly placed within the genus *Viola*. Within the genus, *V. mandshurica* was grouped with several closely related *Viola* species, including *V. seoulensis*, *V. prionantha*, and *V. patrinii*, with strong bootstrap support (Figure 3). The additional ML tree based on concatenated chloroplast protein-coding genes recovered the same close relationship among *V. mandshurica*, *V. seoulensis*, *V. prionantha*, and *V. patrinii*, supporting the phylogenetic placement inferred from the complete-plastome dataset (Figure S2). This result supports the phylogenetic placement of *V. mandshurica* within *Viola*.

## Discussion and conclusion

Among the representative *Viola* plastomes compared in Table S1, V*. mandshurica* is most similar to the closely related species *V. seoulensis*, *V. prionantha*, and *V. patrinii* in genome size, LSC/SSC/IR lengths, GC content, and gene content. This conserved organization is consistent with previous reports showing that plastome structure is generally stable within *Viola* (Cheon et al. 2019; Cao et al. 2022). No obvious species-specific gene loss, large structural rearrangement, or marked IR expansion/contraction was detected in *V. mandshurica* based on the present comparison. Therefore, the main value of this plastome lies not in unusual genomic features, but in providing an additional complete plastome resource for a morphologically diverse and taxonomically complex genus. The duplicated *rpl2* and *ndhB* genes in the IR regions and the trans-spliced structure of *rps12* are consistent with common features of land-plant chloroplast genomes and previously reported *Viola* plastomes (Cheon et al. 2019). These results support the reliability of the genome assembly and annotation, and indicate that *V. mandshurica* does not show evident species-specific structural changes in these intron-containing genes. Although its plastome structure is generally conserved, complete chloroplast genome sequences can still provide useful variation in non-coding regions and rapidly evolving loci for species identification and marker development, especially among closely related plant taxa (Dong et al. 2012, 2015).

The phylogenetic analysis based on complete chloroplast genome sequences strongly supported the placement of *V. mandshurica* within *Viola*. In the maximum-likelihood tree, *V. mandshurica* was closely associated with *V. seoulensis*, *V. prionantha*, and *V. patrinii*. This relationship is generally consistent with previous chloroplast-marker and plastome-based studies of *Viola*, including Korean *Viola* taxa and the reported sister relationship between *V. prionantha* and *V. seoulensis* (Yoo and Jang 2010; Duan et al. 2020). It is also supported by comparative plastome analyses in which *V. patrinii* and *V. prionantha* were placed in the same major clade and morphological grouping was broadly congruent with the chloroplast-genome phylogeny (Cao et al. 2022). In addition, the revised classification of *Viola* places these species in sect. *Plagiostigma* subsect. *Patellares* (Marcussen et al. 2022). Given that *Viola* is a large genus with complex morphological variation and unresolved infrageneric relationships, additional plastome resources are useful for improving phylogenetic sampling, testing species boundaries, and resolving relationships among closely related taxa (Marcussen et al. 2022). Complete plastome datasets have also been shown to improve phylogenetic resolution at low taxonomic levels and can support future studies of population structure and conservation genetics (Parks et al. 2009; Hollingsworth et al. 2016).

In conclusion, this study reports the complete chloroplast genome of *V. mandshurica* and clarifies its phylogenetic position within *Viola*. The plastome is structurally conserved relative to closely related species, but it provides a useful genomic resource for future comparative plastomics, chloroplast marker development, species identification, and phylogenomic studies in *Viola* and Violaceae.

## Supplementary Material

Supplemental Material.docx

## Data Availability

The genome sequence data supporting this study are openly available in GenBank of NCBI at https://www.ncbi.nlm.nih.gov under the accession number PZ316350. The associated BioProject, BioSample, and SRA accession numbers are PRJNA1303833, SAMN57403932, and SRR38226385, respectively.
