# Supplementary material for: The complete chloroplast genome of Viola mandshurica (Violaceae) and its phylogenetic analysis
Source: Mitochondrial DNA B Resour. 2026 Jul 8;11(8):930–5. doi: 10.1080/23802359.2026.2699499 (PMC13353473; doi:10.1080/23802359.2026.2699499)
Supplement: Supplemental Material.docx [file TMDN_A_2699499_SM9340.docx]

**The complete chloroplast genome of *Viola mandshurica* (Violaceae) and its phylogenetic analysis**

Feng Ni, Lina Pan, Jingjing Xia, Dujuan Zhan

**Figure S1.** **The distribution of chloroplast genome sequencing depth for *Viola mandshurica* is depicted in the graph, with the horizontal axis representing genomic position and the vertical axis indicating sequencing depth.**


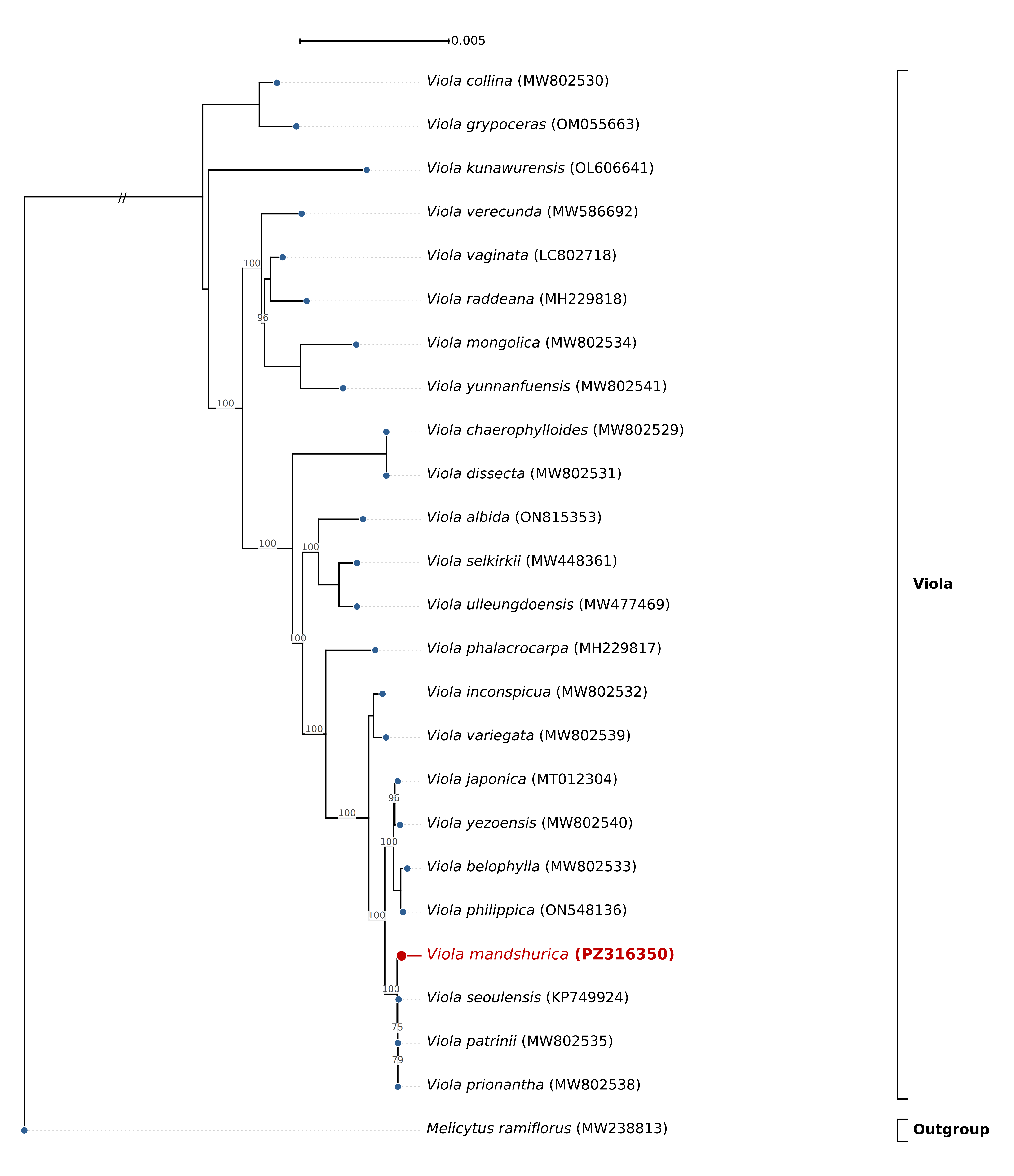


**Figure S2. Maximum-likelihood phylogenetic tree based on concatenated chloroplast protein-coding genes.** A total of 68 shared chloroplast protein-coding genes were extracted from the sampled plastomes of 24 *Viola* species and the outgroup *Melicytus ramiflorus* (MW238813). Each gene was aligned separately using MAFFT, and the resulting alignments were concatenated into a 62,884 bp supermatrix containing 4,569 variable sites and 1,026 parsimony-informative sites. Maximum-likelihood analysis was performed using IQ-TREE 2, with the best-fit model TVM+F+R4 selected by ModelFinder according to the Bayesian information criterion. Branch support was assessed with 1000 ultrafast bootstrap replicates, and bootstrap values ≥70% are shown at the nodes. The newly sequenced *V. mandshurica* (PZ316350) is highlighted in red. The double slash indicates that the branch leading to the outgroup was shortened for visualization. The PCG-based tree recovered the same close relationship among *V. mandshurica*, *V. seoulensis*, *V. prionantha*, and *V. patrinii* as observed in the complete-plastome phylogeny.

**Figure S3.** **The cis-splicing genes in the chloroplast genome of *Viola mandshurica* are depicted in a schematic map using CPGView.** The exons of the cis-splicing genes are represented in black, while the introns are shown in white. Arrows indicate the sense direction of genes. It should be noted that the lengths of exons and introns are not drawn to scale.

**
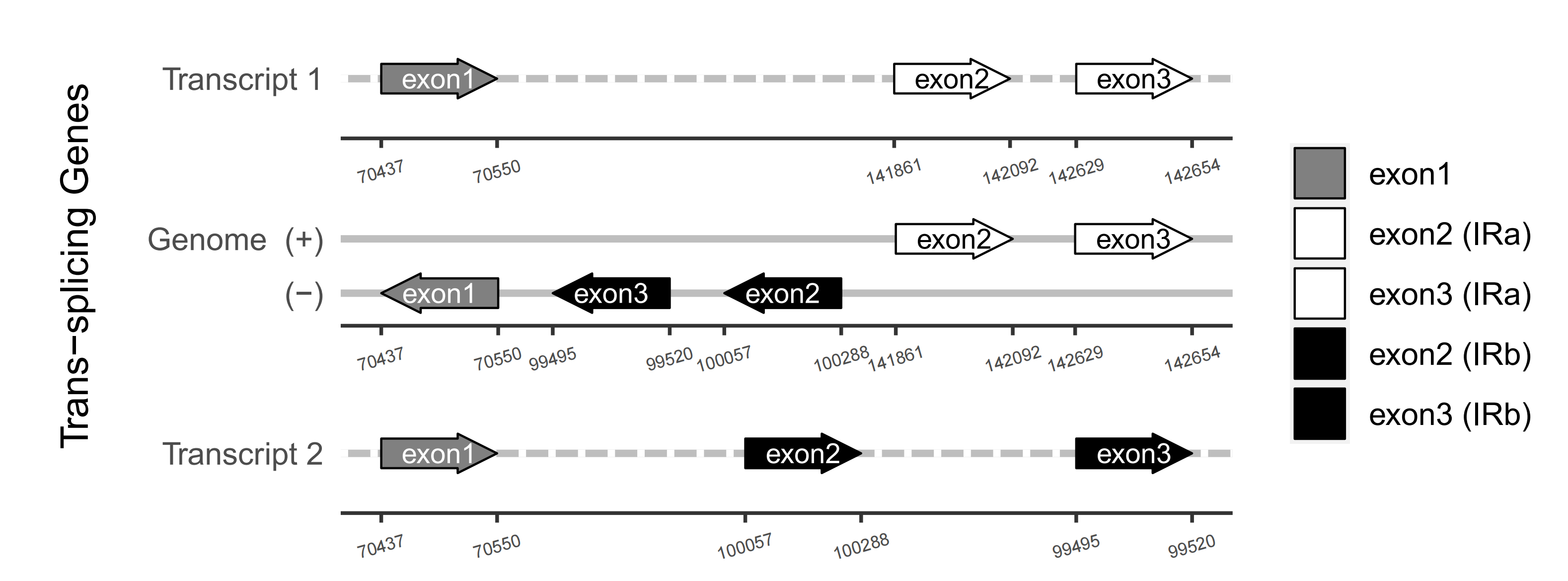
**

**Figure S4.** **The trans-spliced gene *rps12* in the chloroplast genome of *Viola mandshurica* is depicted in a schematic map using CPGView.** An arrow indicates the sense direction of the gene. It should be noted that the lengths of exons are not drawn to scale.

**Table S1. Comparative chloroplast genome features of *Viola mandshurica* and nine *Viola* species**

| **Species** | **Accession** | **Genome size (bp)** | **GC (%)** | **LSC (bp)** | **IR (bp)** | **SSC (bp)** | **Unique genes (PCGs/tRNAs/rRNAs)** |
| --- | --- | --- | --- | --- | --- | --- | --- |
| *Viola mandshurica* | PZ316350 | 156,476 | 36.30 | 85,672 | 26,400 | 18,004 | 112 (78/30/4) |
| *Viola seoulensis* | KP749924 | 156,507 | 36.30 | 85,691 | 26,404 | 18,008 | 112 (78/30/4) |
| *Viola prionantha* | MW802538 | 156,509 | 36.30 | 85,693 | 26,404 | 18,008 | 110 (76/30/4) |
| *Viola patrinii* | MW802535 | 156,508 | 36.30 | 85,694 | 26,404 | 18,006 | 110 (76/30/4) |
| *Viola yezoensis* | MW802540 | 156,537 | 36.27 | 85,706 | 26,417 | 17,997 | 110 (76/30/4) |
| *Viola belophylla* | MW802533 | 156,580 | 36.27 | 85,726 | 26,423 | 18,008 | 110 (76/30/4) |
| *Viola variegata* | MW802539 | 156,582 | 36.29 | 85,769 | 26,411 | 17,991 | 110 (76/30/4) |
| *Viola inconspicua* | MW802532 | 156,624 | 36.30 | 85,822 | 27,122 | 16,558 | 110 (76/30/4) |
| *Viola chaerophylloides* | MW802529 | 157,113 | 36.34 | 85,577 | 27,154 | 17,228 | 110 (76/30/4) |
| *Viola collina* | MW802530 | 157,890 | 36.28 | 86,495 | 27,102 | 17,191 | 110 (76/30/4) |

Gene counts are shown as unique genes; values in parentheses indicate protein-coding genes, tRNA genes, and rRNA genes, respectively.
